# Reply to Grossman et al.: The case for a time-varying oxygen isotopic composition of seawater and cooler Ordovician oceans

**DOI:** 10.1073/pnas.2426540122

**Published:** 2025-03-17

**Authors:** Nivedita Thiagarajan, Aivo Lepland, Uri Ryb, Trond H. Torsvik, Leho Ainsaar, Olle Hints, John Eiler

**Affiliations:** ^a^Division of Geological and Planetary Sciences, California Institute of Technology, Pasadena, CA 91125; ^b^Section for Marine Geology, Geological Survey of Norway, Trondheim 7040, Norway; ^c^Department of Geology, University of Tartu, Tartu 50411, Estonia; ^d^Department of Geology, Tallinn University of Technology, Tallinn 19086, Estonia; ^e^The Fredy & Nadine Herrmann Institute of Earth Sciences, The Hebrew University of Jerusalem, Jerusalem 9190401, Israel; ^f^Department of Geosciences, University of Oslo, Oslo 0316, Norway

Grossman et al. (1) echo a longstanding debate (2, 3): Do the isotopic compositions of ancient marine carbonates reflect past surface temperatures, past ocean compositions, or postdepositional diagenesis? Carbonate-clumped isotope geochemistry adds a constraint, yet the question remains.

The answer must first explain why the δ^18^O values and apparent temperatures from clumped isotopes of ancient carbonates are more variable than modern and recent equivalents. Figure 1 of Grossman et al. (1) provides an example, where reconstructed seawater δ^18^O in narrow time intervals of the Paleozoic, including the Ordovician, span an ~8‰ range, vs. the ~1 to 2‰ range inferred from similar materials deposited over the last several million years (4).

Historically, there have been three approaches to this problem: to either average across otherwise unexplained variability (the approach taken by ref. 1), to assert that one can use criteria such as carbonate textural type or trace metal content to pick samples unaffected by burial history (also a part of ref. 1’s argument), or to assert that the lowest measured apparent temperature from clumped isotopes has fortuitously captured the original depositional composition (5, 6).

We (7) argue that none of these approaches are sound, nor do they yield consistent results. This is obvious from Figure 1 of the comment (1). If brachiopods are the “winners” of the diagenetic sweepstakes then why do their reconstructed δ^18^O_seawater_ vary so much over narrow periods of time? (see also ref. 3). If this variability reflects some component of diagenetic change as is generally recognized; (8, 9), then how could it help to average across a group of changed and hopefully unchanged samples?

We (7) propose an alternative approach: Accept the complete dataset while acknowledging diagenetic influence and use the patterns of correlated change in clumped isotope apparent temperature, δ^18^O_carbonate_, and derived δ^18^O of waters to recognize the forms and extents of diagenetic modification (10). This method allows us to constrain the limits on possible depositional temperature and seawater δ^18^O. While external constraints remain necessary—we suggest δ^18^O_seawater_ should vary minimally with latitude within narrow time intervals and change only gradually by ~1‰ per 100 My (11)—our approach neither depends on exceptional carbonate preservation nor assumes specific water–rock ratios during alteration.

Grossman et al. point out, there is only 1 point with a δ^18^O_seawater_ of −4‰. However our dataset includes 19 samples with δ^18^O_seawater_ values below −1‰ (approximately equal to a Cenozoic ocean at times with no polar ice sheets) and 38 samples with a δ^18^O_seawater_ below 0‰ (the value suggested by ref. 1). Our findings indicate a systematic pattern of low δ^18^O_seawater_ beyond isolated extreme values. We remain convinced that our approach is a sounder foundation to stand on than the past practices.

Finally, we do not believe Grossman et al.’s argument regarding paleontological constraints on the possible ranges of marine temperature are sound. Palaeozoic tabulate corals and stromatoporoids have become extinct; absent direct comparisons with modern surviving species, it is unknown whether they could survive temperatures within our modeled temperature ranges.
